# Predicting acute kidney injury in cancer patients using heterogeneous and irregular data

**DOI:** 10.1371/journal.pone.0199839

**Published:** 2018-07-19

**Authors:** Namyong Park, Eunjeong Kang, Minsu Park, Hajeong Lee, Hee-Gyung Kang, Hyung-Jin Yoon, U. Kang

**Affiliations:** 1 Department of Computer Science and Engineering, Seoul National University, Seoul, Republic of Korea; 2 Department of Internal Medicine, Seoul National University College of Medicine, Seoul, Republic of Korea; 3 Department of Biomedical Engineering, Seoul National University College of Medicine, Seoul, Republic of Korea; 4 Internal Medicine, Seoul National University Hospital, Seoul, Republic of Korea; 5 Pediatrics, Seoul National University Children’s Hospital, Seoul, Republic of Korea; University of Sao Paulo Medical School, BRAZIL

## Abstract

How can we predict the occurrence of acute kidney injury (AKI) in cancer patients based on machine learning with serum creatinine data? Given irregular and heterogeneous clinical data, how can we make the most of it for accurate AKI prediction? AKI is a common and significant complication in cancer patients, and correlates with substantial morbidity and mortality. Since no effective treatment for AKI still exists, it is important to take timely preventive measures. While several approaches have been proposed for predicting AKI, their scope and applicability are limited as they either assume regular data measured over a short hospital stay, or do not fully utilize heterogeneous data. In this paper, we provide an AKI prediction model with a greater applicability, which relaxes the constraints of existing approaches, and fully utilizes irregular and heterogeneous data for learning the model. In a cohort of 21,022 cancer patients who were registered into Korea Central Cancer Registry (KCCR) in Seoul National University Hospital between January 1, 2004 and December 31, 2013, our method achieves 0.7892 precision, 0.7506 recall, and 0.7576 F-measure in predicting whether a patient will develop AKI during the next 14 days.

## Introduction

Acute kidney injury (AKI) is a common complication in cancer patients [1, 2]. A study on a cohort of 37,267 Danish cancer patients [3] reports that the 1-year and 5-year risk of AKI were 17.5% and 27.0%, respectively, with the 1-year risk increasing to 31.8% to 44.0% among patients with multiple myeloma, liver cancer, and kidney cancer. Also, during the intensive care unit (ICU) stay, 12% to 49% of critically ill cancer patients experience AKI, with 9% to 32% of them requiring renal replacement therapy (RRT) [4, 5]. AKI in patients with cancer is strongly associated with substantial morbidity and mortality [1, 2, 6]. Previous studies report a three-month mortality rate of greater than 30% among cancer patients with AKI [7], and a six-month mortality rate of 73% in critically ill cancer patients with AKI [8]. Moreover, development of AKI may decrease the likelihood that cancer patients receive optimal cancer treatment as the dose of chemotherapy is often required to be reduced, or potentially curative medication may have to be contraindicated for patients with cancer [3, 4, 7]. Despite decades of research, there does not exist an effective therapy for AKI yet, with the only supportive measure being RRT [9]. As a result, it is of great importance to take timely preventive measures, and reduce the risk of developing AKI.

In this paper, we aim to propose a predictive model for AKI. More specifically, the focus of our work is to develop a highly general AKI prediction model for irregular and heterogeneous clinical data available at the time of prediction. Although several studies have been conducted for predicting AKI [10–14], the scope and applicability of existing methods have been limited. Many models make predictions of whether a patient will develop AKI using data measured during one hospital stay [10, 12, 14], or assume ICU settings where patients are under constant, close monitoring [10, 13, 15]; clinical data obtained in these settings are usually regular both in terms of frequency and the type of measurements taken. However, about two thirds of AKI are acquired in the community (i.e., outside of hospitals) [16], in which case the measurements are relatively infrequent and much less regular. Also, several existing predictive models [12, 17–19] are not designed with various, heterogeneous clinical data in mind. Some of them perform predictions based on the value of a single variable such as estimated glomerular filtration rate (eGFR) [17] and neutrophil gelatinase-associated lipocalin (NGAL) [19]; and many methods do not fully use non-temporal variables and temporal variables (i.e., variables that are measured repeatedly over time as in the case of serum creatinine (SCr) for patients experiencing kidney function decline) for prediction.

Our proposed method addresses the aforementioned limitations of existing approaches. First, our method does not make predictions only for the duration when patients are hospitalized; instead, it assumes outpatient treatments, which enables predictions for a greater number of patients. Second, our approach does not assume that clinical data are regular and frequently observed as in ICU environment, but supposes a more relaxed condition where data can be irregular and infrequently obtained as is often the case in less intensive treatments. Third, our method is designed to fully utilize available heterogeneous data; both non-temporal and temporal data are used in making predictions. Our approach is to build a machine learning model that learns from irregular and heterogeneous data taken over a possibly longer period of time than a single hospital stay, predict the maximum SCr value within the next 14 days, and use the predicted SCr value for predicting the occurrence and severity of AKI.

The main contributions of this paper are as follows:

- **Model.** We describe our model for AKI prediction that relaxes the constraints of existing prediction models, and thus has an improved applicability.
- **Method.** We present a detailed explanation of our method. In particular, we delineate how we preprocess the heterogeneous and irregular data, how we create feature vectors, and how we structure our proposed framework for AKI prediction.
- **Experiment.** We provide extensive empirical evidences for the performance of our approach, obtained with cross validation on the medical records of 21,022 cancer patients.

## Methods

### Ethics statement

The protocol of this study was in accordance with the principles expressed in the Declaration of Helsinki. The institutional review board at SNUH approved this study (H-1509-051-702), and waived the necessity to obtain informed consent from patients since the study design was innocuous to the patients.

### Detecting and staging AKI

**Fig 1:**
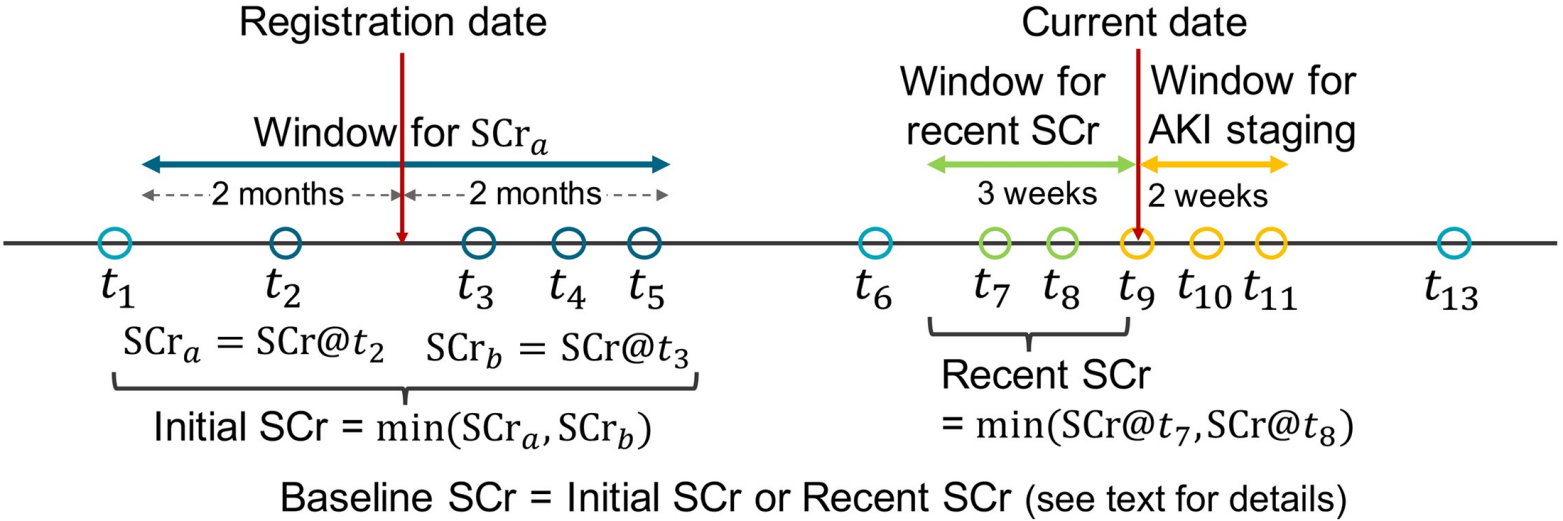
Definitions and time windows for serum creatinine (SCr) measurements used for detecting and staging AKI. Circles represent SCr measurements of a single patient taken at time points *t*_1_, ⋯, *t*_13_. Double-headed arrows represent time windows. The first window is centered around the registration date of a patient, and used to obtain SCr_*a*_. The second and third windows are positioned next to each other such that the current date is located between them, and they are used for obtaining recent SCr, and staging AKI, respectively. See text for more details on SCr_*a*_, SCr_*b*_, and baseline SCr.

Several classification systems [20–22] have been developed for the diagnosis of AKI. Among them, Kidney Disease: Improving Global Outcomes (KDIGO) [20] has been widely used for detecting and staging AKI. How we detect AKI occurrences and calculate their stages in this paper is based on the KDIGO criteria. Our approach uses several different definitions and time windows for SCr. Fig 1 provides an illustration of these definitions and windows for SCr. Below we describe these definitions, and how they are used in detail.

Two different SCr values are used for the diagnosis and staging of AKI: the initial SCr and the recent SCr. Let SCr_*a*_ denote the first one among all SCr values measured between two months before and after the registration date, and let SCr_*b*_ denote the first SCr value measured after the registration. The initial SCr is defined to be:

1. The smaller value of SCr_*a*_ and SCr_*b*_, if both exist, or
2. SCr_*a*_ or SCr_*b*_ otherwise.

The recent SCr is defined as the minimum SCr value among those measured within the past three weeks.

#### Detecting AKI

The normal range of SCr is 0.8-1.3 mg/dL in adult males, and 0.6-1.1 mg/dL in adult females. AKI is determined to have occurred if either of the following two cases is satisfied:

- The current SCr is greater than 1.5 times the initial or the baseline SCr; or
- The current SCr is greater than the initial or the baseline SCr by more than 0.3 mg/dL.

#### Staging AKI

There are three AKI stages for patients diagnosed as having AKI, from stage 1 to stage 3; a higher AKI stage indicates greater severity, leading to an increased risk for death and need for RRT. In addition, we use AKI stage 0 for patients without AKI.

We define the baseline SCr to be the SCr value that led to the detection of AKI with respect to the current SCr; according to the rules for AKI detection, baseline SCr is either the recent SCr of current SCr or the initial SCr. For each AKI occurrence, we go over the SCr value corresponding to the AKI incidence, and the following SCr values measured within the next two weeks (if available); for the current SCr (the one currently being visited), we compute its ratio (= each SCr / baseline SCr) and its increment (= each SCr − baseline SCr) with respect to the baseline SCr to compute the AKI stage as follows.

- If the ratio of the current SCr to baseline SCr is greater than or equal to 3.0, stage 3.
- If the current SCr is greater than or equal to 4.0 mg/dL, stage 3.
- For all computed ratios, we determine the stage by ratio to be
  - 3 if the maximum ratio is greater than or equal to 3.0,
  - 2 if the maximum ratio is smaller than 3.0, and greater than or equal to 2.0,
  - 1 if the maximum ratio is smaller than 2.0, and greater than or equal to 1.5,
  - 0 otherwise.
  For all computed increments, we determine the stage by increment to be
  - 1 if the maximum increment is greater than 0.3 mg/dL,
  - 0 otherwise.
  The greater one among the stage by ratio and the stage by increment becomes the AKI stage.

#### Comparison with KDIGO

Our approach is different from KDIGO in a few respects. First, our criteria do not include urine output. Considering that, clinically, AKI diagnosis among cancer patients has been mostly performed using serum creatinine (SCr) measurements instead of urine output, we assume it will not make much difference to not use urine output. Also, our dataset consists of records of cancer patients, while urine output is rarely measured routinely for cancer patients; therefore, it is not possible to apply AKI criteria that are based on urine output. As a result, we use SCr measurements for AKI detection and staging.

Second, the size of the window used by our criteria for AKI detection is different from that used in KDIGO. While KDIGO diagnoses AKI by checking an increase in SCr with respect to the baseline value within the prior 7 days, our criteria compare the current SCr with the recent SCr which is obtained using a window of three weeks, or with the initial SCr which can be away from the current SCr by more than three weeks. This decision is because our study considers not just inpatients whose SCr values can be measured every day or every 2–3 days, but both inpatients and outpatients. In particular, the interval between SCr measurements of outpatients ranges from 2–3 weeks to 3–6 months, depending on the types of treatments performed. We use a window of three weeks to take this into account, and make the most of available SCr data.

Third, while KDIGO includes the initiation of RRT as a criterion for stage 3 AKI, it is not used in our criteria as we had limited access to the records on RRT. Instead, we exclude patients who had severe chronic kidney disease when hospitalized, which we detail in the Data preprocessing subsection.

### Data description

The Ministry of Health and Welfare initiated a hospital-based nationwide cancer registry called Korea Central Cancer Registry (KCCR) in 1980 in order to accurately measure, and build a database of the incidence of cancer nationwide. Our data include patient information measured and collected by Seoul National University Hospital (SNUH), which is a tertiary referral hospital in Seoul, Korea, having more than 1,000 general admission beds. In particular, our dataset consists of several irregular and heterogeneous temporal and non-temporal data measured from cancer patients who registered for KCCR for the first time in SNUH between January 1, 2004 and December 31, 2013. Cancer treatments were performed after patients were registered into KCCR, after which follow-up care was given at different timing for each patient. In the following subsections, we give a description of non-temporal and temporal data.

#### Non-temporal data

Non-temporal data consist of several different types of medical records as follows: date-type variables (birth date, dialysis date, etc), Boolean-type variables (sex, whether a patient has diabetes, tuberculosis, and chronic liver disease, etc), and numerical/categorical-type variables (age, weight, height, systolic and diastolic blood pressure, occupation, hemoglobin, protein, sodium, triglyceride, etc). In total, a single entry in the non-temporal data consists of 57 variables. In the non-temporal data, each patient has only one entry, i.e., these 57 variables are measured once for each patient. Non-temporal variables were measured the first time patients were admitted to the hospital during the four months between two months before and after the registration date. Since cancer patients undergoing either surgical operations or anticancer treatments routinely receive only basic serum lab testing over the course of their treatment, most biomarkers are not assessed frequently enough to be used as temporal data. Also, blood pressure records are used as non-temporal data, as they are not maintained in the SNUH’s electronic medical record (EMR) system in many cases.

#### Temporal data

In contrast to the non-temporal data, the temporal data can have multiple entries for a single patient, each associated with a specific date; at the same time, there can be no entry for a patient in case no measurements have been taken for that patient. Our temporal data are composed of six different sets of records (SCr measurements, patients’ age at SCr measurements, CT scans, intravenous (IV) chemotherapeutics, oral chemotherapeutics, and nephrotoxic drugs), which we describe below.

#### SCr measurements

SCr measurements are records of all SCr measurements of registered patients, which mainly consist of SCr values and the time when they were measured. Each patient in our dataset can have multiple SCr measurements.

#### Patients’ age at SCr measurements

This is a record of the patients’ age when each SCr measurement was performed.

#### CT scans

CT scans data mainly consist of the date, code, and region of all CT scans taken for each patient. We do not take account of the type and dosage of contrast medium used for CT scans, but consider only whether a patient has taken a CT scan at a given date.

#### IV chemotherapeutics

IV chemotherapeutics data contain the following information: the data of prescription, drug code, drug name, unit of injection, and duration in days. For the sake of simplicity, we consider only whether a patient is administered IV chemotherapeutics at a specific date, but do not take the unit of injection into account.

#### Nephrotoxic drugs

This is a prescription history of nephrotoxic drugs with the following information: drug code, start and end dates of medication, unit and amount of prescribed drugs. To take the effect of nephrotoxic drugs into account, we group them into two groups, the first being diuretics drugs, and the second being ACE (Angiotensin Converting Enzyme) inhibitor and ARB (Angiotensin Receptor Blocker). We consider whether a patient is prescribed a nephrotoxic drug at a specific date separately for each of these two groups.

#### Oral chemotherapeutics

Oral chemotherapeutics data are a history of oral chemotherapeutic agents that are dispensed both inside and outside hospitals, which contains the date of prescription, drug code, number of days of medication, and the total amount.

### Data preprocessing

We preprocess the given dataset to make it available as an input to the regression algorithms, and maximize their predictive performance. First, we exclude some variables or some user’s data based on the following criteria:

- We exclude those patients who were receiving maintenance dialysis, and had an initial estimated glomerular filtration rate (eGFR) value of less than 15.0 ml/min per 1.73 m^2^, as this signifies that these patients had already been suffering from severe chronic kidney disease (CKD) at the time of hospital admission. The CKD-EPI formula [23] is used in calculating eGFR values.
- We filter out the following variables since they do not provide meaningful information for AKI prediction: occupation, income level, educational status, follow-up duration, and whether a patient experienced tuberculosis or chronic liver disease.
- We exclude variables, such as whether a patient had hemodialysis, peritoneal dialysis, continuous renal replacement therapy (CRRT), kidney transplantation (KTPL), that are related with dialysis as they are directly associated with AKI.
- We exclude redundant variables, such as whether a patient received a cancer surgery or a chemotherapy, took a computerized tomography (CT) scan, or had a dialysis, since they can be inferred from other variables and thus add no new information for AKI prediction.
- We also exclude such variables whose missing rate is greater than 0.4 to reduce errors that can be introduced by imputation, which is performed after ruling out variables.

Second, while variables with high missing rates are excluded in the previous step, remaining variables may still have missing entries. Supposing that the regression models that we employ do not allow missing entries, we perform multiple imputation for the missing values of the remaining variables. Since the decision to measure biomarkers is based on existing patient records, and data are not missing completely at random in general, we assume that missing values in our dataset are missing at random; under this assumption, we apply multivariate imputation by chained equations (MICE) [24] to the non-temporal data of entire patients. We use the *fancyimpute* library [25] for the implementation of MICE. With the parameters set to their default values, missing values are imputed 100 times, and averaged to produce the final estimate.

Third, while some of our data are originally numeric values, others are written in text such as male/female and yes/no. We convert these non-numeric values into numbers so that the dataset consists of only numeric values that can be fed into the regression models.

### Creating feature vectors and target values

**Fig 2:**
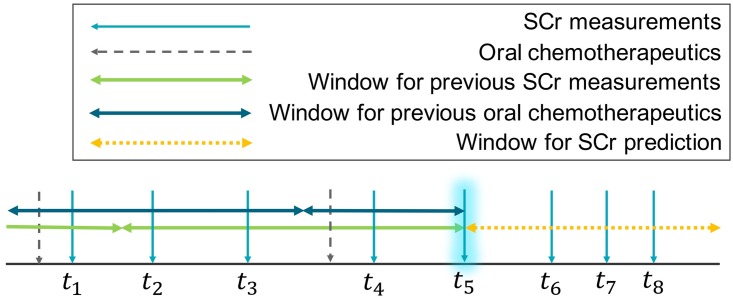
Window-based data creation. Blue, vertical arrows represent serum creatinine (SCr) measurements, and grey, vertical arrows in a dashed line represent oral chemotherapeutics. There are other temporal data that are also included as temporal features, but we omit them for simplicity’s sake. *t*_1_, ⋯, *t*_8_ represent the times when SCr values are measured. The highlighted blue arrow at *t*_5_ indicates the current SCr. The double-headed arrows represent windows: the windows to the left of time *t*_5_ are to summarize the temporal data before the current time *t*_5_, and the window to the right of time *t*_5_ is to capture the target value.

Using the preprocessed data, we create a set of feature vectors and target values. A feature vector and a target value are created for one SCr measurement, and a feature vector consists of non-temporal and temporal features. Fig 2 gives an overview of how we create temporal features and target values using a window-based approach.

#### Non-temporal features

All non-temporal variables in the preprocessed dataset become the non-temporal part of a feature vector. Therefore, if a patient took several SCr measurements, the same non-temporal part appear repeatedly over all feature vectors created for this patient. We try to balance the ratio between non-temporal and temporal features by excluding non-temporal variables that are not meaningful or do not add much new information for AKI prediction.

#### Temporal features

We aim to incorporate the changes of temporal variables over different time periods into the temporal part of a feature vector by using a window-based approach. For each temporal feature of the feature vector, we define separate windows, and compute the summary or statistics of the measurements that fall within each window. We use [*s*, *e*) notation to denote a window whose relative start (inclusive) and end (exclusive) times with respect to the current moment are *s* and *e* (both in hours), respectively. For example, [−7 × 24, −1 × 24) indicates a window for measurements taken from seven days ago (inclusive) to yesterday (exclusive). Temporal variables included in the feature vector are as follows.

- Current SCr value for which the feature vector is created.
- Patient’s age at the time of current SCr measurement.
- Statistics of previous SCr values. We use two windows: [−180 × 24, −30 × 24), [−30 × 24, 0). We compute the average, standard deviation, minimum, maximum, delta (maximum − minimum) of previous SCr values contained in each window. We also compute delta-delta values (defined as the difference between delta values from two consecutive windows). In case no previous SCr values exist for any of the given window, we do not create a feature vector for the current SCr measurement.
- Number of previous CT scans. We use two windows: [−45 × 24, −15 × 24), [−15 × 24, 0).
- Number of injections of intravenous chemotherapeutics. We use two windows: [−45 × 24, −15 × 24), [−15 × 24, 0). We distinguish the nephrotoxicity of anticancer drugs into ‘yes’, ‘no’, and ‘unknown’, and calculate the injection count separately.
- Whether non-anticancer, nephrotoxic drugs are dosed. We use one window: [−30 × 24, 0). Since the prescription length of nephrotoxic treatments is shorter than three to four weeks in many cases, we use a shorter window for this variable than those used for the above ones. We count (1) whether angiotensin receptor blockers (ARBs) or angiotensin-converting enzyme (ACE) inhibitors are dosed, and (2) whether diuretics are dosed within the window.
- Whether oral chemotherapeutics are dosed. We use one window: [−30 × 24, 0). While the prescription length of oral chemotherapeutics is usually slightly longer than that of nephrotoxic drugs, we use a relatively short window for this variable as the prescription length varies a lot, depending on the patients’ condition, and the treatment regimen. We distinguish the nephrotoxicity of oral chemotherapeutics into ‘yes’, ‘no’, and ‘unknown’, and count separately.

#### Target value

Our goal is to predict the maximum SCr value that will occur in a relatively short period of time. More specifically, among SCr values contained in a target window, we use the maximum as the target value for the given feature vector. We use [1, 14 × 24) as the target window. If there exist no SCr values in the target window, we skip the current SCr measurement, and do not create a corresponding feature vector.

#### Post-processing

We apply squeezing and normalization to the feature vectors for post-processing. First, we compute the lower and upper bounds for each feature, and set the value of those that lie outside of the boundary to the boundary value. This is to reduce the impact of outlier values. We set 2% and 98% of each feature as the lower and upper bound. Second, we normalize each column of (squeezed) feature vectors to prevent those columns that have large values compared to others from dominating the regression performance. We perform z-score normalization, which is to standardize each column by subtracting the mean from it and scaling it to unit variance.

### AKI prediction and performance evaluation

Using the feature vectors and target values, we train linear and non-linear regression models that predict SCr values. Once we have the trained regressors, we can predict the maximum SCr value that will occur within the [1, 14 × 24) window. Given that the windows for AKI detection and AKI prediction are of the same size, we apply our criteria for AKI detection and staging to the obtained maximum SCr value to predict the occurrence and stage of AKI, which we then compare with the corresponding ground truth which is obtained using the actual records of SCr measurements that belong to the same two-week period.

#### Using the inverse of SCr

While we use SCr values in the temporal part of a feature vector and the target value, we also allow our method to use the inverse of SCr. While we use SCr as the target value to represent the condition of overall renal function, the value of SCr and the kidney function are known to be inversely proportional, that is, their relationship is non-linear. Using the SCr value is a straightforward choice for the goal of predicting the SCr value; however, using the inverse of SCr might make the regression more accurate, especially in the case of linear regression models. If we use 1/SCr in feature vectors, we use the minimum 1/SCr as the target value, and use the inverse of what the regressors return in predicting AKI occurrence and stage.

#### Regression models and settings

The following linear and non-linear regression models are used in our experiments.

- **Linear regression models:** linear regression, ridge regression, lasso, least-angle regression (LARS), stochastic gradient descent (SGD)
- **Non-linear regression models:** random forest, multivariate adaptive regression splines (MARS)

In order to prevent overestimation of model performances, we use different datasets for training and testing the model. In particular, we use *k*-fold cross validation (CV), where the entire dataset is divided into *k* subsets, and the model is trained using *k* − 1 subsets and tested using the other one subset. The CV process is repeated *k* times, using a different subset as the test data each time; then the *k* test results are averaged to produce a single estimation. Most of these regression models have hyperparameters that need to be optimized, such as the *α* parameter of Ridge regression model which controls the regularization strength. We also use *k*-fold CV in order to choose model parameters that maximize prediction performance over the validation set.

Since using the same validation data for tuning hyperparameters and evaluating model performance biases the model to the validation set, and may thus result in an optimistic performance estimation [26], we use nested *k*-fold CV where the inner CV is used to tune the model parameters and choose the best model, and the outer CV is used to evaluate the model selected by the inner CV. In our experiments, we set *k* to 3 for both the inner and outer CV. With this setting, the inner CV receives two thirds of the data split by the outer CV, and divides it into three subsets, among which two subsets are used for training a model with specific hyperparameters, and one subset is used for model validation.

We use *scikit-learn* [27] for all regression models, except for MARS for which we use *py-earth* library [28].

## Results

### Dataset

The number of patients initially included in our dataset is 91,970. After applying the steps for preprocessing data, and creating feature vectors and target values, the non-temporal and temporal data used in the experiment consist of the following number of records:

- Non-temporal data (number of patients): 21,022
- Temporal data
  - SCr measurements: 988,808
  - CT scans: 238,573
  - IV chemotherapeutics: 645,098
  - Nephrotoxic drugs: 7,528
  - Oral chemotherapeutics: 81,552

**Fig 3:**
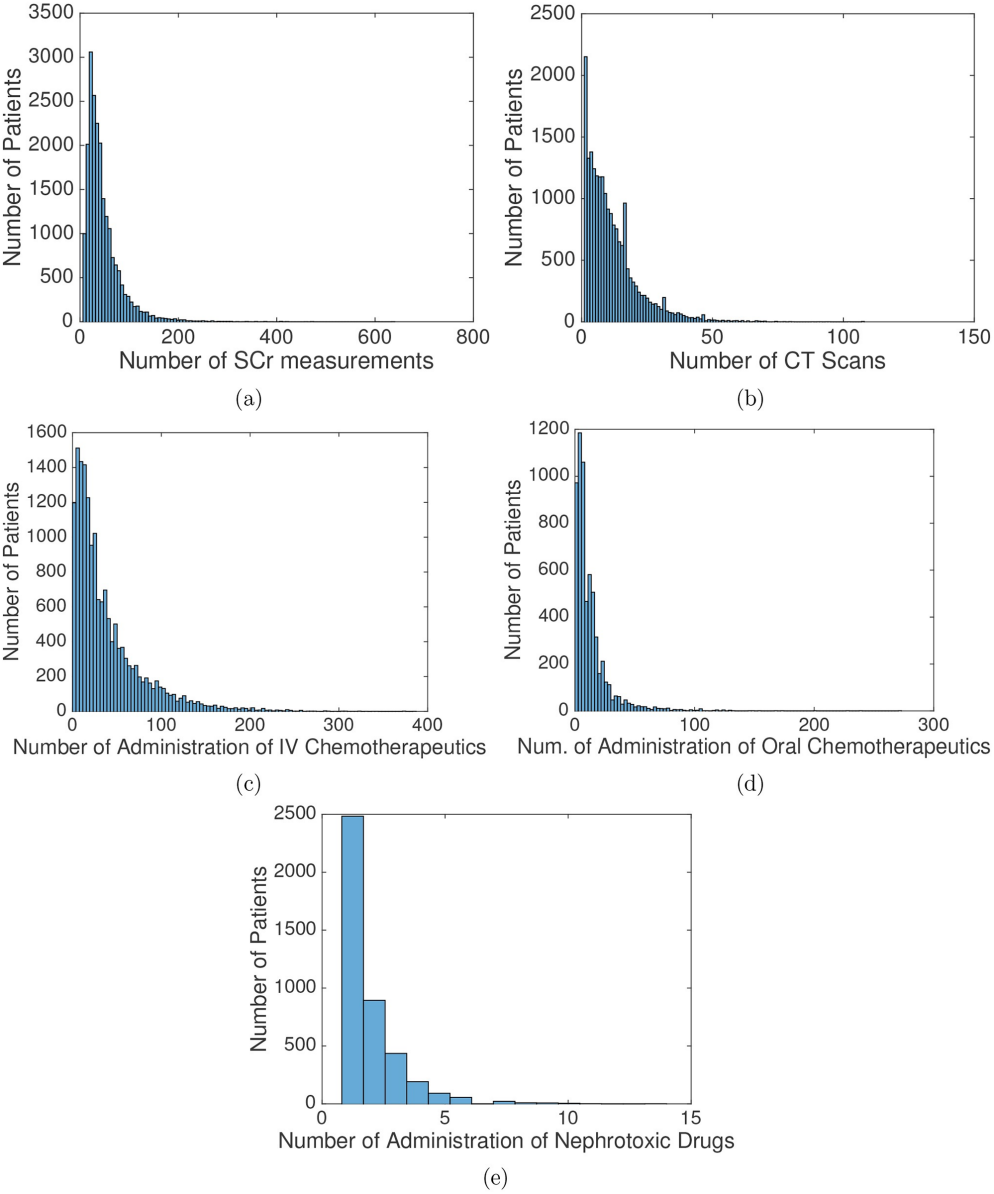
Distribution of number of temporal records. (a) Serum Creatinine (SCr) measurements, (b) CT scans, (c) administration of intravenous (IV) chemotherapeutics, (d) administration of oral chemotherapeutics, and (e) administration of nephrotoxic drugs.

Table 1 lists the descriptive statistics of non-temporal variables where the median and interquartile range are shown for non-categorical variables, and the number of patients in each category is shown for categorical variables. The distribution of patients by cancer type is shown in Table 2: about 30% of the patients had respiratory tract cancer, and patients with respiratory tract cancer, gastrointestinal tract cancer, and thymus cancer comprised more than 60% of the total patients. In Table 3, we present the missing rate of non-temporal variables. The distribution of the number of temporal records among the included patients is shown in Fig 3.

**Table 1:** Descriptive statistics of non-temporal variables: The median and interquartile range are shown for non-categorical variables, and the number of patients in each category is shown for categorical variables.

Variable	median (interquartile range) or # patients in each category	Variable	median (interquartile range) or # patients in each category
Chronic liver disease Y/N	N: 18,682, Y: 2,340	Calcium	9.1 (8.7–9.4)
CRRT Y/N	N: 20,522, Y: 500	Phosphate	3.5 (3.1–3.9)
CT scan Y/N	Y: 20,339, N: 683	Blood urea nitrogen	14 (11–17)
Number of CT scans taken	9 (4–15)	Glucose	102 (91–121)
Diabetes Y/N	N: 18,478, Y: 2,544	Systolic blood pressure	121 (110–133)
HD Y/N	N: 20,723, Y: 299	Diastolic blood pressure	76 (70–83)
Hypertension Y/N	N: 16,320, Y: 4,702	Uric acid	4.7 (3.7–5.8)
KTPL Y/N	N: 20,985, Y: 37	Sodium	140 (138–141)
PD Y/N	N: 21,021, Y: 1	Potassium	4.2 (4–4.5)
Tuberculosis Y/N	N: 20,109, Y: 913	Chloride	104 (102–106)
Age at diagnosis	59 (50–67)	Total carbon dioxide	26 (24–28)
Birth date	49/05/01 (41/06/01–58/10/01)	Smoking Y/N	N: 12,454, Y: 3,251, n/a: 5,317
Cancer code	See Table 2.	Body weight	61.65 (54.7–69.1)
Chemotherapy Y/N	Y: 17,380, N: 3,642	Height	163.4 (157.1–169)
Clinical study participation Y/N	N: 19,991, Y: 1,031	Body mass index	23.2947 (21.1556–25.4003)
Dialysis Y/N	N: 20,306, Y: 716	Educational status	High school: 5,035, university: 4,085, elementary school or lower: 2,682, middle school: 2,204, graduate school or higher: 852, etc: 170, illiterate: 91, n/a: 5,903
Sex	M: 12,623, F: 8,399	Occupation	Unemployed: 4,866, homemaker: 3,558, self-employment: 1,770, company employee: 1,520, etc.: 318, professionals: 199, student: 126, soldier: 60, n/a: 8,605
Surgery Y/N	Y: 11,135, N: 9,887	Income level	No answer: 2,426, middle: 2,193, low: 733, high: 167, n/a: 15,503
The first eGFR value	82.5952 (69.7669–94.8359)	Radiation treatment Y/N	Y: 4,633, N: 44, n/a: 16,345
Platelet	227 (166–288)	Triglyceride	105 (75–148.75)
White blood cell	6.4 (5–8.2)	Low-density lipoproteins	44 (34–54)
Hemoglobin	13 (11.5–14.2)	Mean number of smoking days per year	25 (15–40)
Cholesterol	168 (142–196)	High-density lipoproteins	101 (76–126)
Alanine transaminase	21 (14–37)	Date of dialysis	11/06/26 (09/03/20–14/02/25)
Aspartate transaminase	23 (18–38)	Date of the first CRRT	11/04/28 (09/03/19–13/10/12)
Alkaline phosphatase	77 (60–105)	Date of the first HD	12/03/15 (09/03/17–15/02/28)
Albumin	4 (3.6–4.3)	Date of KTPL	10/09/01 (05/05/18–14/10/01)
Bilirubin	0.7 (0.5–1.1)	Date of the first PD	08/04/03 (08/04/03–08/04/03)
Total protein	7.1 (6.6–7.5)		

CT: Computerized tomography, HD: hemodialysis, CRRT: Continuous renal replacement therapy, PD: peritoneal dialysis, KTPL: kidney transplant, eGFR: estimated glomerular filtration rate, and AKI: Acute kidney injury.

**Table 2:** Distribution of patients by cancer type.

Cancer	Number of patients	Cancer	Number of patients
Respiratory tract cancer	6,871 (32.68%)	Skin cancer	230 (1.09%)
Gastrointestinal tract cancer	4,237 (20.16%)	Endocrine cancer	184 (0.88%)
Thymus cancer	2,405 (11.44%)	Soft tissue cancer	128 (0.61%)
Hematologic malignancy	1,763 (8.39%)	Mesothelioma	91 (0.43%)
Breast cancer	1,656 (7.88%)	Metastasis of unknown origin (MUO)	59 (0.28%)
Female genitourinary organ cancer	1,188 (5.65%)	Bones and joint cancer	58 (0.28%)
Genitourinary tract cancer	908 (4.32%)	Melanoma	51 (0.24%)
Central nervous system cancer	440 (2.09%)	Kaposi’s sarcoma	18 (0.09%)
Head and neck cancer	387 (1.84%)	Merkel-cell carcinoma	8 (0.04%)
Male genitourinary organ cancer	340 (1.62%)		

**Table 3:** Missing rate of non-temporal variables.

Variable	Missing rate	Variable	Missing rate	Variable	Missing rate
Chronic liver disease Y/N	0.0000	Platelet	0.2113	Chloride	0.3519
CRRT Y/N	0.0000	White blood cell	0.2113	Total carbon dioxide	0.3519
CT scan Y/N	0.0000	Hemoglobin	0.2113	Smoking Y/N	0.4116
Number of CT scans taken	0.0000	Cholesterol	0.2172	Body weight	0.4150
Diabetes Y/N	0.0000	Alanine transaminase	0.2175	Height	0.4151
HD Y/N	0.0000	Aspartate transaminase	0.2177	Body mass index	0.4172
Hypertension Y/N	0.0000	Alkaline phosphatase	0.2180	Educational status	0.4299
KTPL Y/N	0.0000	Albumin	0.2182	Occupation	0.5335
PD Y/N	0.0000	Bilirubin	0.2183	Income level	0.7738
Tuberculosis Y/N	0.0000	Total protein	0.2196	Radiation treatment Y/N	0.8891
Age at diagnosis	0.0000	Calcium	0.2367	Triglyceride	0.8913
Birth date	0.0000	Phosphate	0.2369	Low-density lipoproteins	0.8981
Cancer code	0.0000	Blood urea nitrogen	0.2369	Mean number of smoking days per year	0.9067
Chemotherapy Y/N	0.0000	Glucose	0.2576	High-density lipoproteins	0.9393
Clinical study participation Y/N	0.0000	Systolic blood pressure	0.2746	Date of dialysis	0.9874
Dialysis Y/N	0.0000	Diastolic blood pressure	0.2747	Date of the first CRRT	0.9924
Sex	0.0000	Uric acid	0.2772	Date of the first HD	0.9935
Surgery Y/N	0.0000	Sodium	0.3450	Date of KTPL	0.9990
The first eGFR value	0.1253	Potassium	0.3455	Date of the first PD	0.9999

CT: Computerized tomography, HD: hemodialysis, CRRT: Continuous renal replacement therapy, PD: peritoneal dialysis, KTPL: kidney transplant, eGFR: estimated glomerular filtration rate, and AKI: Acute kidney injury.

### Regression accuracy

We measure how accurately our method predicts the serum creatinine (SCr) using several regression models, and how using SCr and the inverse of SCr affects its accuracy. In order to measure the regression accuracy, we need to compare the predicted target value against the actual measurement. Since regressors return only the predicted value without the exact time of the prediction, for the sake of comparison, we assume that the predicted SCr value is measured at the same time as the target value, that is, the maximum SCr or the minimum 1/SCr in the target window.

**Fig 4:**
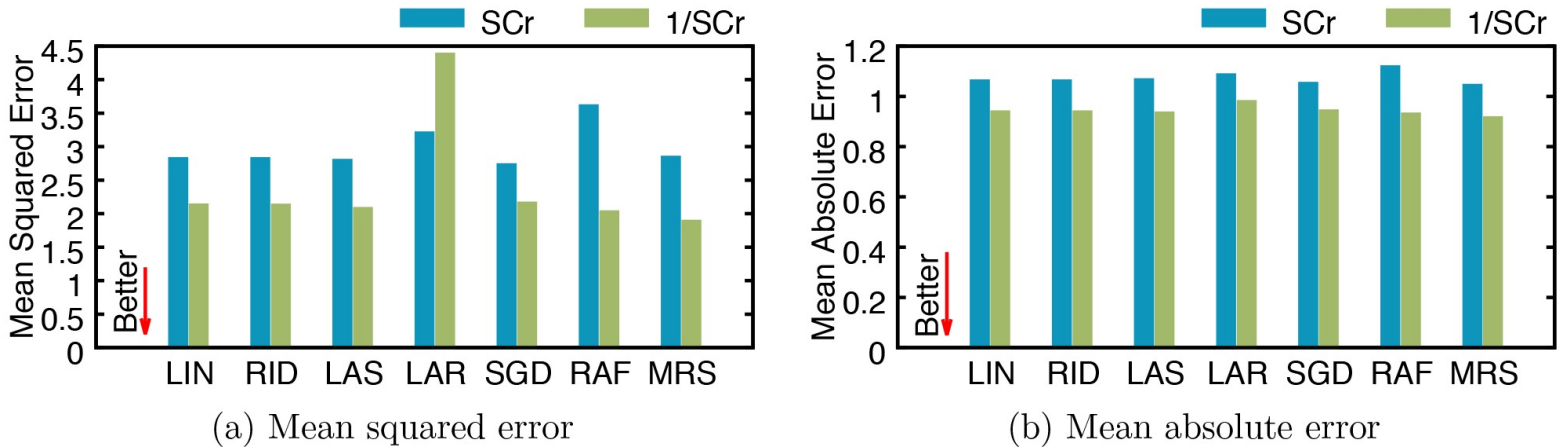
Performance of serum creatinine (SCr) regression by several models using SCr and 1/SCr. (a) mean squared error, and (b) mean absolute error. LIN: linear regression, RID: ridge regression, LAS: lasso, LAR: least-angle regression (LARS), SGD: stochastic gradient descent, RAF: random forest, and MRS: multivariate adaptive regression splines (MARS).

We report mean squared error (MSE) and mean absolute error (MAE) values obtained from both versions of regression models in Fig 4. In experiments using SCr, MARS demonstrates the best performance (MSE: 2.8386 and MAE: 1.0435) with the average MSE and MAE scores being 2.9713 and 1.0691, respectively. Fig 4 shows the effectiveness of using 1/SCr, which improves the regression performance: MSE and MAE scores are improved in almost all cases, with an average improvement being 19% and 12%, respectively. When using 1/SCr, the average MSE and MAE score are 2.3947 and 0.9388, respectively with MARS showing the best performance (MSE: 1.8839 and MAE: 0.9144).

### Classification accuracy

We measure how effectively our method classifies AKI stages using several regression models, and how using SCr and the inverse of SCr affects its performance. The predicted AKI stages are compared against the ground truth AKI stages obtained with actual SCr measurements.

**Fig 5:**
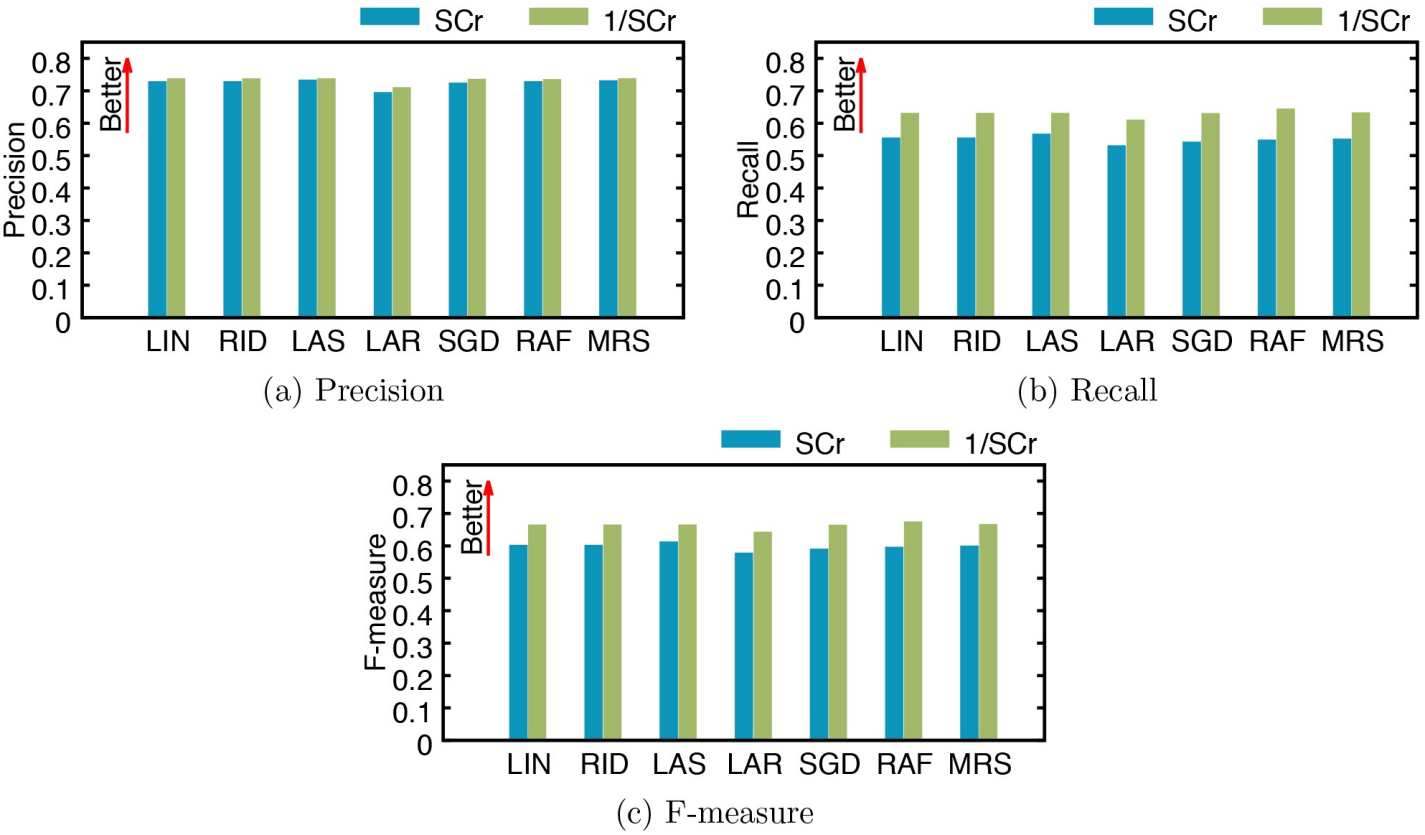
Performance of AKI stage classification by several models using SCr and 1/SCr. (a) precision, (b) recall, and (c) F-measure. LIN: linear regression, RID: ridge regression, LAS: lasso, LAR: least-angle regression (LARS), SGD: stochastic gradient descent, RAF: random forest, and MRS: multivariate adaptive regression splines (MARS).

In Fig 5, we report the precision, recall, and F-measure obtained from the two versions of regression models. In experiments where we use SCr, the average precision, recall, and F-measure are 0.7203, 0.5461, and 0.5935, respectively, with lasso showing the best performance (precision: 0.7296, recall: 0.5628, and F-measure: 0.6090). When we use 1/SCr instead of SCr, all models exhibit improved performance across all classification metrics: the average precision, recall, and F-measure are 0.7293, 0.6261, and 0.6594, respectively, and the random forest shows the best performance (precision: 0.7313, recall: 0.6402, and F-measure: 0.6706).

**Fig 6:**
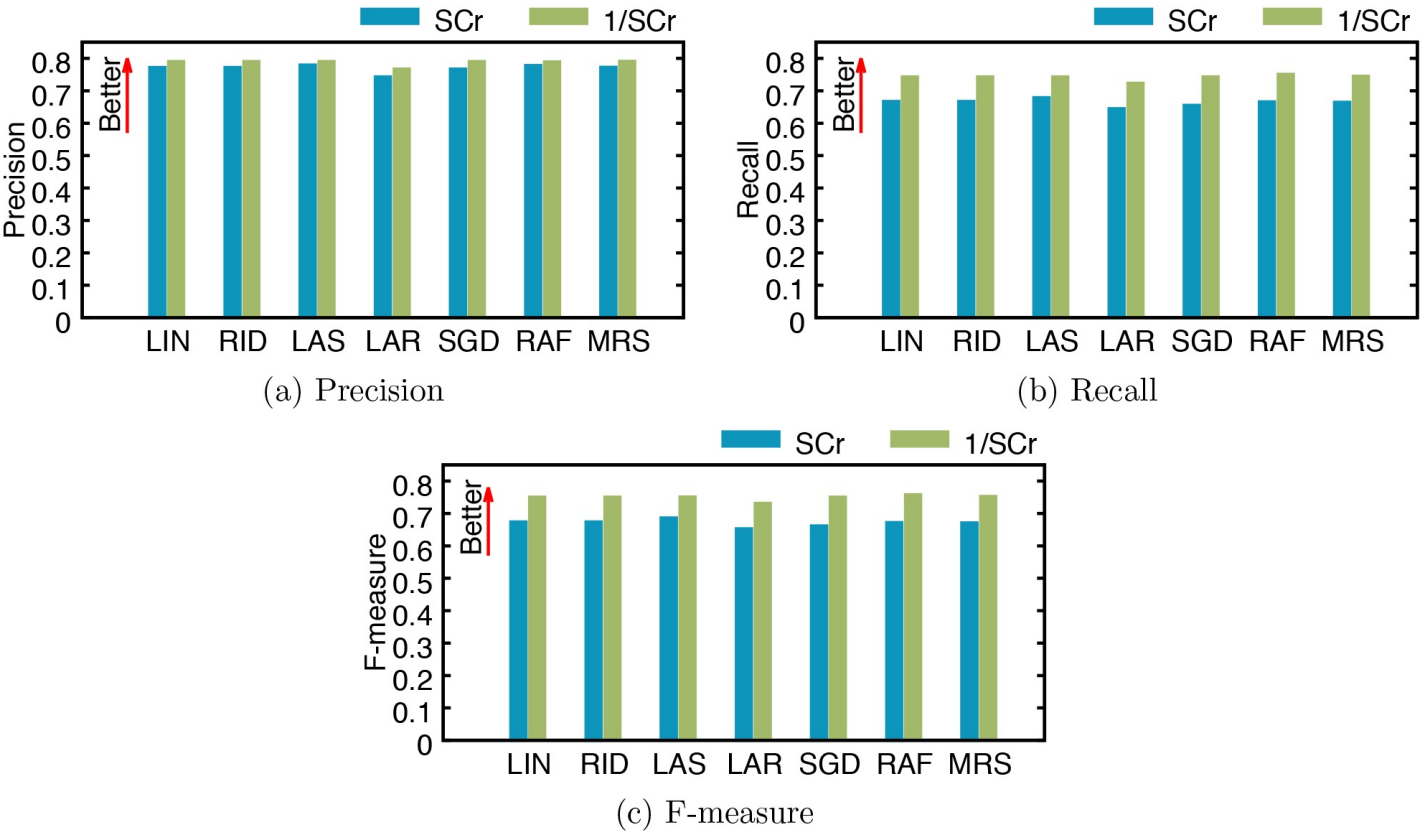
Performance of AKI occurrence classification by several models using SCr and 1/SCr. (a) precision, (b) recall, and (c) F-measure. LIN: linear regression, RID: ridge regression, LAS: lasso, LAR: least-angle regression (LARS), SGD: stochastic gradient descent, RAF: random forest, and MRS: multivariate adaptive regression splines (MARS).

We also report the precision, recall, and F-measure for classifying AKI occurrence in Fig 6. While the overall trend is similar between Figs 5 and 6, scores for all metrics are higher when we classify AKI occurrence than when we classify AKI stages; classifying AKI occurrence is an easier problem than classifying AKI stages since classifying into four AKI stages is reduced to a simpler task of classifying into AKI non-occurrence and occurrence (which correspond to AKI stage 0, and AKI stages 1 to 3, respectively). When we use SCr, lasso shows the best performance (precision: 0.7794, recall: 0.6790, and F-measure: 0.6862), with the average precision, recall, and F-measure being 0.7692, 0.6633, and 0.6702, respectively. When using 1/SCr, random forest achieves the best performance (precision: 0.7892, recall: 0.7506, F-measure: 0.7576); the average precision, recall, and F-measure are 0.7869, 0.7415, and 0.7490, respectively.

### Model evaluation across years

**Fig 7:**
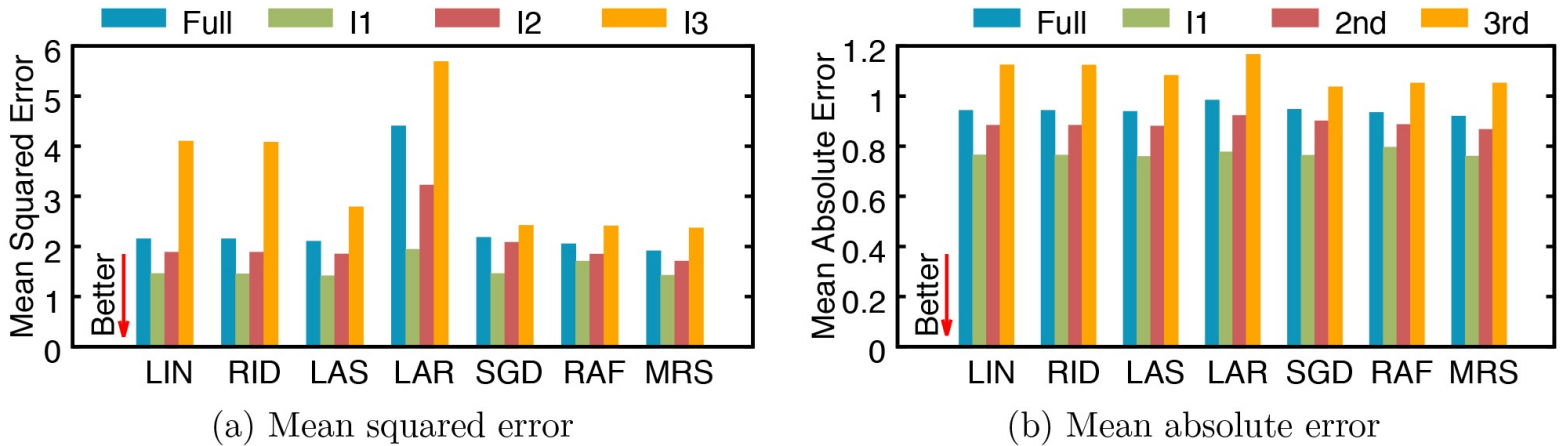
Performance of serum creatinine (SCr) regression by several models trained with the full data and subsets. (a) mean squared error, and (b) mean absolute error. I1: an interval between 2004/1/1 and 2007/11/31, I2: an interval between 2007/12/1 and 2011/10/31, and I3: an interval between 2011/11/1 and 2015/9/30. LIN: linear regression, RID: ridge regression, LAS: lasso, LAR: least-angle regression (LARS), SGD: stochastic gradient descent, RAF: random forest, and MRS: multivariate adaptive regression splines (MARS).

**Fig 8:**
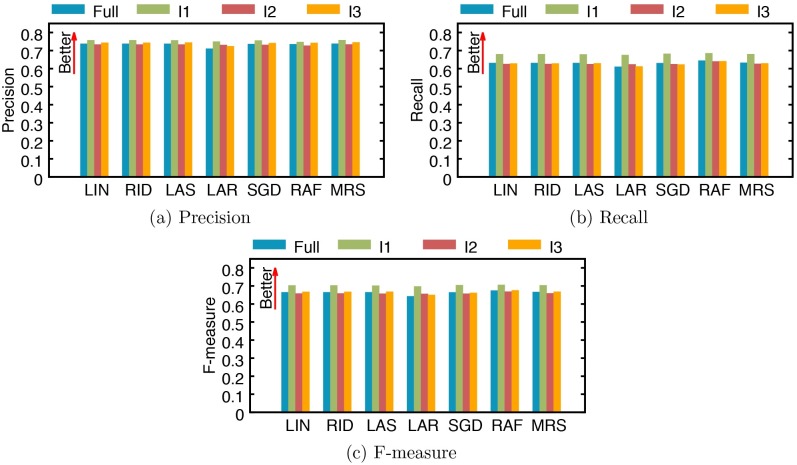
Performance of AKI stage classification by several models trained with the full data and subsets. (a) precision, (b) recall, and (c) F-measure. I1: an interval between 2004/1/1 and 2007/11/31, I2: an interval between 2007/12/1 and 2011/10/31, and I3: an interval between 2011/11/1 and 2015/9/30. LIN: linear regression, RID: ridge regression, LAS: lasso, LAR: least-angle regression (LARS), SGD: stochastic gradient descent, RAF: random forest, and MRS: multivariate adaptive regression splines (MARS).

**Fig 9:**
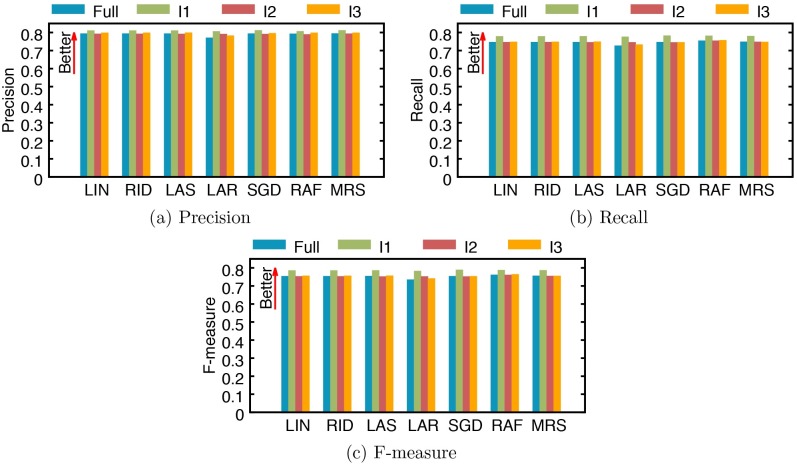
Performance of AKI occurrence classification by several models trained with the full data and subsets. (a) precision, (b) recall, and (c) F-measure. I1: an interval between 2004/1/1 and 2007/11/31, I2: an interval between 2007/12/1 and 2011/10/31, and I3: an interval between 2011/11/1 and 2015/9/30. LIN: linear regression, RID: ridge regression, LAS: lasso, LAR: least-angle regression (LARS), SGD: stochastic gradient descent, RAF: random forest, and MRS: multivariate adaptive regression splines (MARS).

Our dataset was collected in a single university hospital (SNUH) in Seoul for over 10 years. Since the study period is quite long, patients’ care may have changed along the years. In order to measure how the performance of our method changes over time, we divide the data into three subsets using three, equal-sized intervals (I1, I2, and I3) that cover the entire period, where I1 is between 2004/1/1 and 2007/11/31, I2 is between 2007/12/1 and 2011/10/31, and I3 is between 2011/11/1 and 2015/9/30. Then we estimate the regression and classification performances on each subset, as was reported for the entire dataset. For this experiment, we only use 1/SCr. Fig 7 reports the regression performance of all models over the full data and three subsets divided by three intervals (I1, I2 and I3) in terms of mean squared error (MSE) and mean absolute error (MAE). While regression performances across time intervals vary a lot by models and time intervals, it is noticeable that all models achieve the best performance in interval I1. We also report model performances for classifying AKI stages (Fig 8) and occurrences (Fig 9). Overall, the differences between models and time intervals in Figs 8 and 9 are smaller than those in Fig 7, and models perform classification with similar performances across time intervals. The performance of AKI occurrence classification in interval I1 is better than those in other intervals by 1.8% to 4.4% (Fig 9).

## Discussion

The primary goal of our experiments was to evaluate the model performance on predicting whether a patient will develop AKI within the next two weeks, and our approach achieved 0.7892 precision, 0.7506 recall, and 0.7576 F-measure for this task. While some machine learning algorithms were better than others in terms of classification and regression accuracy, their performances were quite close to each other. We could also observe that model performances differed a lot depending on whether SCr or 1/SCr was used.

While there exist several previous studies for predicting AKI, in this paper, we focus on developing an AKI prediction method with a high applicability that makes use of heterogeneous and irregular cancer patients’ data collected over a long period of time, with the goal of enabling better clinical decision making and AKI prevention.

To this end, we assessed the generalizability of our approach by performing nested *k*-fold CV. Considering that the method is not particularly suited to the environment of SNUH, we think that the proposed approach could perform similarly on the data of other hospitals. Furthermore, we ran an experiment to measure how model performances change over 10 years of time, in which we saw that models exhibited almost identical performances across different time periods. It is noticeable, however, that models perform slightly better in interval I1 than in other cases. We hypothesize that this is because for some temporal variables, the average number of records available per feature vector in this period is greater than those in other intervals. For example, there are 11% and 16% more CT scan records on average in interval I1 than in intervals I2 and I3, respectively; also, interval I1 has 18% and 35% more IV chemotherapeutics data than interval I2 and I3. Based on these results, and the fact that the current method can easily be extended to include different types of variables, we envisage an extension of our approach that applies to different clinical populations other than cancer patients in more diverse environments.

### Limitations

There are a few limitations that call for future work. First, our dataset consists of patient records of a single hospital (SNUH) in Seoul. Despite our efforts to do a fair evaluation of our model’s generalizability, we cannot rule out the possibility that our dataset has some characteristics that other datasets do not possess. Therefore, it is our future work to test and improve the model based on the generalizability of our approach on more diverse, external datasets.

Second, our method does not provide procedures to configure windows used for creating feature vectors and target values. While our current settings for these windows are determined based on the variable type and data availability, applying our method to different datasets may require adding new windows, or updating the current settings, in which case users need to provide the number and length of windows used for each variable. It would be helpful to have a principled way for configuring windows.

Third, while our approach considers previous CT scans when creating temporal features by including the number of CT scans, the number of CT scans alone is not sufficient. It would provide much more information for predicting AKI to include the type and dose/volume of contrast medium used for each CT scan, as the nephrotoxicity of one contrast medium might be greater than that of another, and more importantly, the volume of contrast medium may change over the course of treatment. Alternatively, the anatomic region explored by the CT scan could provide an approximate information on the volume of contrast medium used. Unfortunately, both of these information were not available since all related information are maintained separately from the EMR systems that are accessible for research purposes.

Fourth, variables with missing rates greater than 40% were excluded since they can introduce substantial errors that can adversely affect the model performance. However, this also led to the removal of several variables relevant to AKI prediction, which could have resulted in the loss of predictive power.

Fifth, while KDIGO includes urine output and the initiation of RRT as a criteria for AKI staging, they are not used in our criteria due to the lack of those data. More accurate evaluation of AKI staging would be possible if these two variables can be considered along with SCr values.

Sixth, although we provide an assessment of the overall predictive performance of our model, the validity of the variables used by the model remains open to further studies; how different variables interact with each other, and affect the accuracy of AKI prediction in a variety of environments will need to be investigated.

## Related works

Several studies have been conducted for the task of predicting acute kidney injury (AKI). One line of research toward this end has focused on investigating promising biomarkers or employing existing risk models for the prediction and detection of AKI. Haase et al. [19] performed meta-analysis of observational studies to investigate the diagnostic and prognostic power of neutrophil gelatinaseassociated lipocalin (NGAL) as an early biomarker of AKI. Kane-Gill et al. [15] examined what risk factors for AKI are important in elderly patients, and performed regression analyses to see how the significance of risk factors changes in different age groups. Parikh et al. [18] investigated the performance of interleukin-18 (IL-18) as a predictive biomarker for AKI following cardiac surgery. Murugan and Kellum [9] discussed the role of biomarkers for early diagnosis and better prognosis of AKI, and an improved classification system that incorporates novel biomarkers as damage criteria. De Geus et al. [29] and Lameire et al. [30] provided a detailed review of the performance of various biomarkers for the prediction and detection of AKI. Chang et al. [31] studied whether two risk scores for predicting mortality in bypass surgery (STS and ACEF) can be used for AKI prediction after mitral valve repair.

In recent years, machine learning techniques have been increasingly used for AKI prediction. Tirunagari et al. [17] proposed two approaches to detect AKI for patients who are not under constant monitoring condition, such as an ICU; while the focus of [17] is to detect AKI, the one based on the Gaussian process regression can also be used for predicting AKI. In [10] and [11], machine learning algorithms, such as logistic regression, are applied to electronic health record data of pediatric patients obtained from the first 12 hours of ICU stay in order to predict AKI development in the first 72 hours of ICU admission. Kate et al. [12] proposed machine learning models for older adults to predict, at 24 hours from admission, whether a patient will develop AKI later during a hospital stay. Cruz et al. [13] developed a Bayesian network based-model for predicting the risk of developing AKI, which they applied to ICU patient data. Using classification and regression tree (CART), Schneider et al. [14] developed a decision tree for late AKI prediction with the burn patient data obtained from the first 48 hours after admission. Liu et al. [32] proposed a risk model for surgery-related AKI based on preoperative risk factors by utilizing stepwise multivariate logistic regression.

## Conclusion

In this paper, we present our method for predicting acute kidney disease (AKI) from irregular and heterogeneous clinical data. Our contributions are threefold. First, we present a model for AKI prediction that relaxes the constraints of existing prediction models, and thus has a better applicability. Second, we present a detailed explanation of our method. In particular, we delineate how we preprocess the heterogeneous and irregular data, how we create feature vectors, and how we structure our proposed framework for AKI prediction. Third, we provide extensive empirical evidences for the performance of our approach using the dataset of 21,022 cancer patients. Our method achieves 0.7892 precision, 0.7506 recall, and 0.7576 F-measure in predicting whether a patient will develop AKI during the next 14 days.
